# Exploring Marine natural products as potential Quorum sensing inhibitors by targeting the PqsR in *Pseudomonas aeruginosa*: Virtual screening assisted structural dynamics study

**DOI:** 10.1371/journal.pone.0319352

**Published:** 2025-03-28

**Authors:** Manikandan Jayaraman, Vijayakumar Gosu, Rajalakshmi Kumar, Jeyakanthan Jeyaraman, Hak-kyo Lee, Donghyun Shin

**Affiliations:** 1 Structural Biology and Bio-Computing Lab, Department of Bioinformatics, Science Block, Alagappa University, Karaikudi, Tamil Nadu, India; 2 Department of Animal Biotechnology, Jeonbuk National University, Jeonju, Republic of Korea; 3 Mahatma Gandhi Medical Advanced Research Institute, Sri Balaji Vidyapeeth (Deemed to be University), Pillayarkuppam, Puducherry, India; 4 Department of Agricultural Convergence Technology, Jeonbuk National University, Jeonju, Republic of Korea; Kwara State University, NIGERIA

## Abstract

Antibiotic resistance is a critical global health issue, and *Pseudomonas aeruginosa* is a particularly challenging pathogen. This gram-negative bacterium is notorious for its high virulence and resistance to antimicrobial agents, making it a leading cause of nosocomial infections, significantly impacting public health. The adaptability and multidrug resistance of *P. aeruginosa* exacerbate treatment difficulties, resulting in increased morbidity and mortality rates worldwide. Consequently, targeting bacterial quorum sensing (QS) systems is a promising strategy for the development of novel antimicrobial compounds against this resilient pathogen. In this study, a structure-based virtual screening (SBVS) approach was employed to identify marine natural products (MNPs) as potential lead molecules targeting the biofilm-forming PqsR protein of *P. aeruginosa*. A total of ~37,000 MNPs were initially evaluated and ranked based on docking scores using high-throughput virtual screening (HTVS), Standard Precision (SP), and Extra Precision (XP) methods. Ten lead molecules (five from the CMNPD database and five from the MNPD database) were shortlisted based on their docking scores (<−10.0 kcal/mol) and binding free energy values (MM-GBSA ΔG <−40 kcal/mol). Their drug-likeness profiles were assessed using stringent criteria in the QikProp module of Schrödinger, and their chemical reactivity was evaluated through density functional theory (DFT) calculations. The structural and energetic interactions between the identified MNPs and the PqsR-binding pocket were validated through molecular dynamics simulations (MDS) and binding free energy (BFE) calculations. Structural dynamic analyses revealed that the MNP-bound PqsR complexes demonstrated stable interactions within the binding pocket, with hydrophobic residues such as L208, I236, and I263 playing a crucial role in maintaining stability. Among the identified MNPs, CMNPD14329, CMNPD23880, MNPD13399, and MNPD13725 emerged as promising lead molecules for further research. These candidates can serve as foundations for developing structural analogs with enhanced binding affinities for PqsR and other biofilm-forming proteins. Further experimental validation is essential to confirm the therapeutic potential of these identified MNPs.

## 1 Introduction

*Pseudomonas aeruginosa*, a gram-negative pathogen, is a leading cause of hospital-acquired infections and significantly affects immunocompromised patients. It is implicated in a variety of infections, including dermatitis, urinary and respiratory tract infections, with a notable prevalence in individuals with cystic fibrosis [1]. The severity of infections caused by *P. aeruginosa* is exacerbated by its resistance to antibiotics and wide array of virulence factors. These virulence factors encompass the production of potent toxins, extracellular invasive enzymes, and secondary metabolites such as pyocyanin [2]. Additionally, *P. aeruginosa’s* capacity to form resilient biofilms complicates treatment [3]. Due to its substantial threat to public health, the World Health Organization (WHO) has classified *P. aeruginosa* as a “critical-priority” pathogen, highlighting the urgent need for innovative therapeutic strategies [4].

Quorum sensing (QS), also known as cell-cell communication, plays a pivotal role in biofilm formation and antimicrobial resistance in bacteria, rendering it an attractive target for strategies aimed at controlling bacterial pathogenicity and virulence. In *P. aeruginosa*, the coordination of virulence factors and biofilm formation is regulated by the QS system [5]. *P. aeruginosa* employs multiple QS systems, with the LasI/R and RhlI/R systems being the most extensively studied and characterized. These systems are activated by the binding of acyl homoserine lactone autoinducers (AIs), which serve as chemical signals that facilitate communication among bacterial cells [6]. Additionally, there exists a third QS system that relies on alkyl quinolone (AQ) molecules. These three QS systems are intricately interconnected with the Las signal directing the QS pathway, which activates both Rhl and Pqs [7]. The Las system also regulates the expression of the extracellular protease LasB, a zinc metalloprotease with strong proteolytic activity against various tissue substrates that is a key virulence factor in *P. aeruginosa* [8]. The interaction between these QS systems and their downstream effectors reveals a complex regulatory network that drives the pathogenicity and adaptability of *P. aeruginosa*, highlighting potential therapeutic targets for combating *Pseudomonas* infections.

Chemical classes of AIs involved in bacterial QS exhibit significant structural diversity, reflecting the intricate nature of these communication systems. For instance, las and rhl QS systems in *P. aeruginosa* utilize N-acylated l-homoserine lactone derivatives. Conversely, the Pqs system depends on AQ compounds [7]. The biosynthesis of AQs, including crucial signaling molecules such as 2-heptyl-3-hydroxy-4(1H)-quinolone and 2-heptyl-4-hydroxyquinoline, requires a distinct set of enzymes: PqsA, PqsBC, PqsD, PqsE, and PqsH. The process starts with the substrates anthraniloyl-CoA and malonyl-CoA, which are transformed through a series of enzymatic reactions into active AQ signaling molecules [9]. Both 2-heptyl-3-hydroxy-4(1H)-quinolone and 2-heptyl-4-hydroxyquinoline bind to the LysR-type regulator, PqsR, causing conformational changes that trigger a positive feedback loop *via* transcriptional activation of the pqsABCDE operon. This feedback mechanism significantly increases AQ production and regulates the expression of numerous genes involved in virulence and biofilm formation [10]. PqsR is a critical transcriptional regulator in the Pqs system, playing an essential role in the production of Pqs-controlled virulence factors. PqsR-deficient mutants are unable to produce key virulence determinants such as elastase and pyocyanin, underscoring its pivotal role in the pathogenicity and adaptability of *P. aeruginosa* [11]. Recent studies have demonstrated that molecular blockade of PqsR using small-molecule inhibitors can achieve comparable effects. For instance, inhibitors such as vanillin [12] and quinoline‐based derivatives [13] have been reported to effectively disrupt quorum sensing pathways, thereby suppressing the production of virulence factors. These findings highlight the therapeutic potential of PqsR-targeted strategies in mitigating bacterial pathogenicity. Targeting PqsR, specifically quorum-sensing inhibitors (QSIs), is a promising alternative strategy for suppressing *P. aeruginosa* virulence and halting biofilm formation.

Disrupting bacterial communication holds significant promise for reducing the virulence of *P. aeruginosa* and enhancing the efficacy of conventional antibiotics. QSIs represent a ground-breaking approach for combating antibiotic-resistant infections, particularly those involving bacterial biofilms. By interfering with QS, QSIs can potentiate the action of antibiotics, permitting the use of lower doses and minimizing dependence on broad-spectrum antibiotics [14]. A diverse range of natural QSIs has been identified, highlighting their potential to mitigate bacterial virulence and resistance. Compounds such as luteolin [15], coumarin [16], ginseng [17], butein, and sappanol [18] have demonstrated significant anti-quorum sensing activity. In addition to natural QSIs, synthetic compounds have also shown considerable promise. Examples include meloxicam and piroxicam [19], diarylheptanoids [20], benzamide-benzimidazole derivatives [21], and aspirin [22]. However, the clinical application of current QSIs is limited by challenges such as low bioavailability, poor stability, and high toxicity [23]. Addressing these limitations is a critical focus of ongoing research, which aims to develop next-generation QSIs with improved pharmacokinetics, reduced toxicity, and enhanced effectiveness.

In recent years, marine ecosystems have been increasingly recognized as reservoirs of biologically active molecules with significant potential for combating human infectious diseases [24,25]. Among these, marine natural products (MNPs), particularly those derived from microbial organisms, have emerged as promising sources of anti-biofilm agents [26]. Marine microbes constitute an underexplored source of secondary metabolites with potent antimicrobial properties. Owing to their distinct self-defense mechanisms and adaptation to extreme environmental conditions, MNPs encompass a diverse array of chemical compounds. These molecular scaffolds present new and distinctive structures that hold promise for the development of alternative antibiotics effective against biofilms [27–29].

The present computational study aimed to identify potential lead molecules from a diverse array of MNPs housed within two distinct databases: the CMNPD and the MNPD. By leveraging the vast chemical diversity and biological activity data contained in these repositories, we aimed to identify promising candidates for the development of new lead molecules against the therapeutic targets of PqsR from *P. aeruginosa*. This approach not only expands the spectrum of potential bioactive compounds, but also improves the efficiency of drug discovery. By employing advanced computational techniques such as virtual screening, molecular dynamics simulation (MDS), and binding free energy (BFE) calculations, this study screened and evaluated an extensive repository of marine-derived molecules.

## 2 Materials and methods

The SBVS methodology provides an effective and efficient approach for identifying potential lead molecules against PqsR, balancing reasonable accuracy with modest computational effort. Our computational pipeline integrated several advanced methodologies to enhance the screening process. This included an initial virtual screening study to identify promising candidates, followed by the prediction of ADMET properties to assess their drug-likeness, quantum mechanics calculations to delve deeper into the electronic properties of the lead molecules, and structural dynamics studies, incorporating MDS to provide insights into the stability and conformational dynamics of the drug-target complexes. These analyses were further complemented by MM/PBSA binding energy calculations to estimate the free energy of binding, thus enhancing our understanding of the interaction potential and stability.

### 2.1 Structure-based virtual screening

The target protein, PqsR, and its co-inducer molecule (PDB ID: 4JVI) were obtained from the Protein Data Bank (https://www.rcsb.org/structure/4JVI) [30]. This structure underwent meticulous preprocessing steps for the virtual screening studies using the Maestro interface of the Schrödinger software suite (Protein Preparation Wizard, Schrödinger, LLC, New York, NY, 2017-1). The initial step involved adding hydrogen atoms to the structure to ensure its integrity and stability, followed by determination of the ionization states of any heterogeneous groups within the protein using Epik, with the pH set to 7.0 ± 2.0. This step ensured that the ionization states of the protein were accurately represented under physiological conditions. Hydrogen bond assignments were optimized to further refine the protein structure. Subsequently, energy minimization was performed using the Optimized Potentials for Liquid Simulations_3 (OPLS) force field. This process refined the protein structure to ensure an optimal conformation for subsequent docking-based virtual screening studies. The binding-site residues of the target protein were selected based on the native ligand-bound conformation observed in the crystal structure [30]. A receptor grid box encompassing the active site residues was generated to facilitate the docking process. This was accomplished using the ‘Receptor Grid Generation Wizard’ within the GLIDE module. This grid box precisely defines the area of the protein to be targeted in the docking studies, ensuring accurate and efficient virtual screening.

Marine natural products were collected from two databases: CMNPD (https://www.cmnpd.org, ~31,000 compounds) [31] and MNPD (http://docking.umh.es/downloaddb, ~6,000 compounds) [32], with all datasets accessed in September 2022. These databases were specifically chosen for their comprehensive and well-curated collections of marine-derived compounds, renowned for their structural diversity and unique bioactivity. Together, they provide an extensive repository of marine natural products, highlighting their potential for drug discovery and further enhancing the reliability and robustness of our virtual screening process. The chemical accuracy and optimization of the collected MNPs were ensured using the LigPrep module of the Schrödinger suite (LigPrep, Schrödinger, LLC, New York, NY, 2017-1). The optimized ligand datasets were sequentially docked into the binding pocket residues of PqsR using a hierarchical protocol comprising high throughput virtual screening (HTVS), Standard Precision (SP) and extra precision (XP), all with default parameters within the GLIDE module of the Schrödinger suite (Glide, Schrödinger, LLC, New York, NY, 2017-1). At each stage, the top 10% of the highest-ranked compounds were selected and advanced to the subsequent level of screening, ensuring a stringent selection process and enrichment of high-affinity binders for further analysis. Evaluation of the virtual screening results relied on docking scores and binding energies, focusing on docked complexes with a high number of molecular interactions observed during visual inspection, suggesting suitability for further analysis. Additionally, the co-crystallized PqsR ligand was redocked into the binding site to serve as a control molecule. The docking results of this co-crystallized ligand were compared with those of the identified MNPs to assess relative binding efficacy and interaction profiles.

### 2.2 Binding free energy calculation

The MM/GBSA (Molecular Mechanics/Generalized Born Surface Area) method is employed to precisely determine the BFEs of small molecules to biological macromolecules [33]. Using the Prime module (Prime, Schrödinger, LLC, New York, NY, 2017-1), the protein-ligand complex underwent meticulous processing within the MM/GBSA framework. This method integrates the OPLS3 force field and the adaptable Variable-Dielectric Surface Generalized Born solvation model, thereby enhancing accuracy and reliability. The MM/GBSA analysis provides a detailed breakdown of the energy contributions of various factors. These include hydrophobic interactions, van der Waals forces, and solvation effects, with a particular emphasis on the Born electrostatic solvation energy. This thorough analysis showed MM/GBSA as the preferred approach for precisely computing free binding energies (ΔG Bind), offering insights into the molecular interactions governing ligand binding efficacy.

### 2.3 Evaluation of drug-likeness properties and toxicity profile

Preclinical ADMET (absorption, distribution, metabolism, excretion, and toxicity) studies are vital for evaluating drug safety and efficacy. These studies identify pharmacokinetic and toxicological issues early in the drug development process. To optimize costs and streamline the drug development pipeline, various *in silico* methods have been developed to predict and analyze ADMET properties using advanced algorithms and databases. These approaches provide rapid and cost-effective assessments that simulate drug interactions to prioritize compounds with favorable profiles for further experimental validation. The QikProp module of the Schrödinger suite (QikProp, Schrödinger, LLC, New York, NY, 2017-1) was used to evaluate the drug-likeness properties of the identified natural products using the SBVS approach. First, the drug-likeness of all the selected compounds was evaluated using RO5, which identifies compounds with favorable pharmacokinetic properties suitable for oral administration. Additionally, several molecular descriptors were considered to comprehensively assess the suitability of these compounds for further studies. These descriptors included molecular weight (≤500 Da), hydrogen bond donors (0–6), and hydrogen bond acceptors (2–20). MDCK cell permeability was categorized as low for values ≤25 and high for values >500. Parameters such as hERG inhibition (values below −5 indicate potential cardiotoxicity), Caco-2 cell permeability (low for values ≤25 and high for values >500) to assess intestinal absorption, and the brain/blood partition coefficient (−3.0 to 1.2) to evaluate the potential for central nervous system penetration were also analyzed. Additionally, the toxicity profiles of the identified products were analyzed using the ProTox-II pharmacokinetics server (https://comptox.charite.de/protox3/) [34], which accepts the SMILES structural notation of the compounds as input. This method provides a cost-effective and efficient way to assess the potential of drug candidates before advancing to further stages of development and testing.

### 2.4 Frontier molecular orbital (FMO) analysis

The FMO analysis employing DFT calculations was conducted to evaluate the chemical reactivities and stabilities of the identified lead molecules. This comprehensive analysis used the Jaguar Module of the Schrödinger suite (Jaguar, Schrödinger, LLC, New York, NY, 2017-1). The obtained results were visualized in the Maestro interface, facilitating a deeper understanding of the electronic structures and potential reactivities of the molecules. The analysis involved various calculations, including electron density (ED) mapping, molecular electrostatic surface potential (MESP), and frontier orbital calculations such as the highest occupied molecular orbital (HOMO) and lowest unoccupied molecular orbital (LUMO). These computations were performed using Becke’s three-parameter exchange–correlation function combined with Lee-Yang-Parr and a 6-31G^**++^ basis sets. These features collectively aid in identifying the molecular properties, chemical reactivity, and stability of compounds [35,36]. In particular, FMO analysis was used to identify the HOMO and LUMO regions as potential sites for electrophilic and nucleophilic interactions, respectively. Electrons can be donated to the receptor from the HOMO region, whereas they are accepted by the LUMO region. Furthermore, the energy difference between the HOMO and LUMO regions, known as the HOMO-LUMO energy gap (HLG), was calculated to assess the stability and bioactivity of the most active compounds.

### 2.5 Molecular dynamics simulation (MDS) and MM/PBSA

To gain insight into the structural stability of protein-ligand complexes, MD simulations were performed using the GROMACS 5.1.4 suite, incorporating the CHARMM27 force field [37]. This approach facilitates the dynamic exploration of complex behaviors over time, providing insights into their stability and interactions. The ligand topologies were obtained from the SWISSPARAM server (http://www.swissparam.ch/) [38]. Furthermore, the CHARMM27 force field was applied to define protein topologies, ensuring an accurate representation of their structural properties. To construct the protein-ligand complexes, the topologies of both the protein and ligand molecules were merged. Subsequently, each resulting complex was positioned 1 nm from the protein surface within a dodecahedral box to ensure an optimal simulation environment. To mimic physiological conditions, water molecules were introduced *via* the TIP3P (Transferable Intermolecular Potential with 3 Points) model. Additionally, the required number of counter ions (Na^+^ and Cl^–^) were incorporated to neutralize any excess charge within the system, ensuring its overall neutrality. Following this setup, an energy minimization step was executed using the steepest descent algorithm, followed by a conjugate gradient algorithm for 50,000 steps. Subsequently, the system was subjected to two equilibration cycles. First, an NVT ensemble (with constant number of particles, volume, and temperature) was applied for 500 ps at a stable temperature of 300 K. This was followed by the NPT ensemble (constant number of particles, pressure, and temperature), maintaining a pressure of 1 bar. These equilibration steps ensured that the system reached a stable state before proceeding to further analysis. After equilibration, the production run was conducted for 100 ns. Subsequently, an analysis was performed using GROMACS-in-built models of the MD trajectories. Finally, the BFE and energy decomposition of each residue were calculated using 300 frames from 60 to 90 ns of the trajectory of each complex using the g_mmpbsa tool [39]. This protocol was based on our previous research [40–42].

## 3 Results and discussion

Although natural compounds, particularly those derived from marine environments, exhibit vast diversity and abundance, their potential as anti-biofilm agents targeting QS proteins remains relatively underexplored. Nevertheless, their extensive and diverse chemical footprint holds significant promise for novel therapeutic applications. In drug design and development, systematic screening of natural products sourced from marine environments against validated druggable targets has become a well-established strategy [35,43,44]. This process was further enhanced by *in silico* methodologies, including the prediction of pharmacokinetic properties and the study of structural dynamics through MD simulations and BFE calculations. These computational techniques provide valuable insights into the design, optimization, efficacy, and safety of potential drug candidates, thereby streamlining the drug discovery pipeline and increasing the probability of identifying effective anti-biofilm agents to combat biofilm-related infections and other medical conditions.

### 3.1 Virtual screening to identify potential marine natural products

SBVS is a widely recognized and commonly used technique in drug design and development. It plays a crucial role in identifying potential inhibitors that target various druggable proteins, particularly those associated with biofilm-related infections, thus aiding in the development of effective treatments to combat bacterial biofilms and associated diseases [45–47]. This study utilized SBVS to identify potential MNPs targeting PqsR, a critical regulator of *P. aeruginosa* biofilm formation. Datasets from two databases including CMNPD and MNPD were screened for PqsR-binding pocket residues. This approach facilitated the exploration of optimal docking conformations for each MNP, thereby identifying the lead molecules that offer the most effective binding interactions with the PqsR protein. Crystal structure of the PqsR co-inducer-binding domain from *P. aeruginosa* complexed with inhibitor 3NH2-7Cl-C9QZN (S1 Fig) was employed for SBVS using the GLIDE module of the Schrödinger software suite. The residues required for GRID generation were determined based on the binding interactions of the inhibitor molecule, enabling precise modelling of the receptor site for effective virtual screening. The hierarchical virtual screening methodology (HTVS→SP→XP) identified ten potential lead molecules: five from the CMNPD (CMNPD4682, CMNPD14329, CMNPD28977, CMNPD23880, and CMNPD24734) and five from the MNPD (MNPD9355, MNPD9492, MNPD9493, MNPD13399, and MNPD13725) (S2 Fig). These hits demonstrated satisfactory docking and binding energy scores as well as favorable molecular interaction profiles with PqsR (Table 1).

**Table 1:** Comparative analysis of molecular interaction profiles of MNPs identified by virtual screening against a control molecule.

MNPs	GLIDE Docking Score (kcal/mol)	MM/GBSA binding energy score (kcal/mol)	Molecular interactions		
**H-bond interaction**	**Stacking interaction**	**Salt bridge interaction**
Control Molecule	−8.20	−39.36	Leu207	-	-
CMNPD4682	−11.59	−56.98	Gln194, Leu197, Leu208 and Ile236,	-	-
CMNPD14329	−11.80	−55.55	Gln194, Leu197, Leu208 and Ile236	-	-
CMNPD28977	−11.85	−52.67	Ile186, Ser196, and Arg209	-	-
CMNPD23880	−12.60	−60.41	Gln194, Leu197, Leu208 and Ile236	-	-
CMNPD24734	−13.08	−56.29	Gln194, Leu207, and Arg209	Tyr258	-
MNPD9355	−12.57	−43.13	Gln194, Leu208 and Ile236	-	-
MNPD9492	−13.07	−64.51	Gln194, Leu197, Leu207 Leu208 and Ile236	Tyr258	-
MNPD9493	−12.68	−64.53	Gln194, Leu197, Leu207 Leu208 and Ile236	Tyr258	-
MNPD13399	−12.44	−64.37	Gln194, Leu197, Leu208 and Ile236	-	-
MNPD13725	−11.88	−56.80	Gln194, Leu197, Ile236, and Tyr258	-	-

### 3.2 Molecular interaction profile of identified marine natural products with PqsR

**Fig 1:**
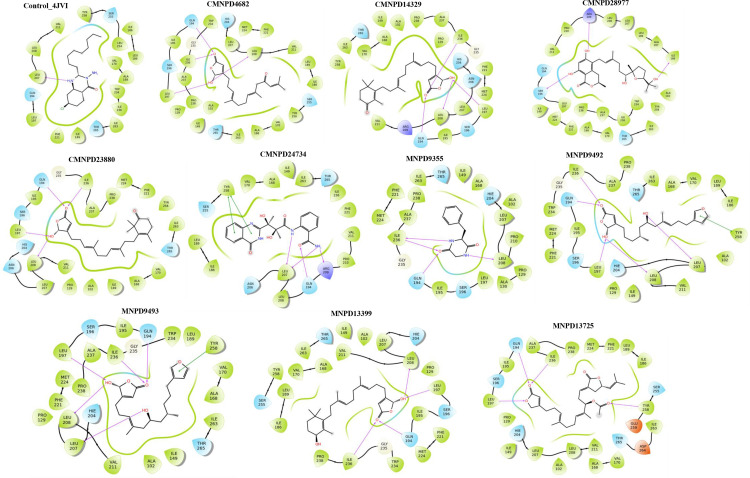
Molecular interaction profile of identified MNPs from the CMNPD and MNPD along with the control molecule. Salt bridges are represented by blue or red lines, while hydrogen bonds are shown with purple arrows. The diagrams also distinguish different types of residues: polar amino acids (sky blue), negatively charged residues (orange), positively charged residues (blue), and hydrophobic amino acids (green).

To gain a better understanding of the molecular interactions between the identified lead molecules and PqsR, the interaction profiles of the complexes were analyzed. The detailed analysis revealed that the main stabilizing interactions between the lead molecules and the PqsR protein were hydrogen bonds and hydrophobic contacts. These interactions are essential for maintaining the stability and affinity of the compounds within the binding pocket, highlighting their potential effectiveness as inhibitors. Initially, the re-docking of the reference inhibitor molecule with PqsR yielded a docking score of −8.2 kcal/mol and an MM/GBSA binding energy score of −39.36 kcal/mol. This redocking revealed molecular interactions similar to those observed in the crystal structure, notably including a hydrogen bond interaction between residue Leu207 and the reference inhibitor molecule (Fig 1). Additionally, similar hydrophobic interactions involving residues Tyr258, Val170, Ile236, Thr265, and Leu208 were observed in the docked complexes of PqsR with the control inhibitor.

The lead molecules from the CMNPD demonstrated a higher number of molecular interactions, and improved docking and MM/GBSA binding energy scores compared to the control molecule (Table 1). Specifically, the docked complexes of CMNPD4682, CMNPD14329, and CMNPD23880 (Fig 1) with PqsR formed four hydrogen bonds (H bonds) within the network. In these complexes, the carbonyl group (C=O) established H-bonds with the polar residue Gln194 and the hydrophobic residue Ile236. The remaining H-bonds involve the hydrophobic residues Leu197 and Leu208 within these complexes. Moreover, CMNPD28977 demonstrated three H-bonds with different residues of PqsR: the hydrophobic residue Ile186, the polar residue Ser196, and the charged residue Arg209, with contact distances of 2.12 Å, 2.36 Å, and 2.2 Å, respectively. Compared to other complexes and the control molecule, the CMNPD24734-PqsR complex exhibited a higher number of molecular interactions, including four H-bonds and two stacking interactions, along with a notable docking score of −13.08 kcal/mol. These intricate molecular interactions underscore the diverse and potent binding capabilities of these compounds for PqsR, suggesting their potential as effective inhibitors.

Furthermore, the docked conformations of the top lead molecules identified through virtual screening against the MNPD revealed substantial intermolecular interactions with the binding pocket residues of PqsR. Four key interactions were identified in the docked complexes of MNPD9355 and MNPD13399, including hydrogen bonding with the binding pocket residues Gln194, Leu208, and Ile236 of PqsR (Fig 1). Notably, Leu197 also contributed to the stabilization of the complex formed by MNPD13399 along with the aforementioned residues. Moreover, in the complexes of MNPD9492 and MNPD9493 with PqsR, five hydrogen bonds were formed involving the residues Gln194, Leu197, Leu207, Leu208, and Ile236. These complexes also exhibited stacking interactions with Tyr258, further stabilizing binding. Similarly, the MNPD13725-PqsR complex demonstrated strong structural stability with the formation of four hydrogen bonds, highlighting the reliability of these interactions.

An MM/GBSA binding energy analysis was employed as a post-docking scoring method to evaluate the binding affinity between MNPs and PqsR (Table 1). Initially, the binding affinity of the control molecule within the PqsR binding pocket was assessed, yielding an MM/GBSA binding energy score of −39.36 kcal/mol. In comparison, the MNPs from the CMNPD database exhibited significantly higher negative binding energy values, ranging from −52.67 to −60.41 kcal/mol, while those from the MNPD database ranged from −43.13 to −64.53 kcal/mol, both indicating stronger binding affinities than the control molecule. Among the compounds from the CMNPD, CMNPD23880 displayed the highest MM/GBSA binding energy, suggesting the most stable interaction with the PqsR protein, whereas within the MNPD, MNPD9492 and MNPD9493 exhibited the highest binding energy, indicating a particularly strong binding affinity. These results underscored the potential efficacy of the identified lead molecules as potent PqsR inhibitors.

### 3.3 Pharmacokinetic profile assessment

Understanding pharmacokinetic profiles is essential for evaluating the characteristics of chemical scaffolds in biological systems. Pharmacokinetic attributes, including RO5 violations, blood-brain barrier impermeability, and HOA, play a pivotal role in this assessment (Table 2 and S2 Table). These physicochemical properties and medicinal chemistry profiles are indispensable for the development of lead molecules as potential drug candidates. Notably, all identified lead molecules exhibited molecular weights below 500 Da, which is a favorable indicator of target accessibility. Notably, none of the lead molecules violated the RO5, further validating their potential as viable drug candidates. The HOA value is a crucial factor in evaluating the bioavailability of a drug, providing insights into its ability to be absorbed into the bloodstream after oral administration. In this context, among the candidates, CMNPD23880 stood out as particularly promising, with an HOA value of 100%, and was classified as class 3, confirming its potential for further drug development endeavors. Additionally, the hydrogen bond donor and acceptor ranges for all the compounds were within the acceptable limits of 0–4 and 5–8, respectively. Furthermore, all the compounds demonstrated favorable CaCo-2 and MDCK cell permeability values, indicating good absorption and permeability profiles. In particular, CMNPD23880 exhibited high CaCo-2 and MDCK cell permeability, suggesting a significant oral bioavailability advantage. Collectively, these properties underscore the potential efficacy and suitability of these molecules as drug candidates.

**Table 2:** Pharmacokinetic profile assessment of MNPs identified by virtual screening through QikProp analysis in Schrödinger software.

MNPs	Molecular Weight	Rule of Five Violation	Donor HB	Accept HB	HOA classification with percentage	QPlogPo/w	QPlogHERG	QPPPMDCK	QPPCaCo	QPlogBB
CMNPD4682	332.4	Nil	1	6.7	91.24 (High)	3.130	−4.835	168.94	370.01	−1.558
CMNPD14329	400.5	Nil	1	6.7	95.17 (High)	4.173	−4.818	124.91	279.88	−1.722
CMNPD28977	448.5	Nil	3	6.7	90.13 (High)	3.889	−4.635	78.11	181.28	−1.888
CMNPD23880	400.5	Nil	1	6.7	100 (High)	4.343	−4.931	209.46	451.53	−1.504
CMNPD24734	396.4	Nil	4	8	63.06 (Medium)	1.856	−5.460	57.43	136.40	−1.678
MNPD9355	204.22	Nil	2	5	62.61 (Medium)	-0.481	−0.718	270.56	141.29	−0.560
MNPD9492	362.4	Nil	2	6.9	92.87 (High)	3.387	−5.793	171.97	376.23	−1.810
MNPD9493	362.4	Nil	2	6.9	92.20 (High)	3.359	−5.765	160.43	352.80	−1.834
MNPD13399	402.5	Nil	2	6.4	96.41 (High)	4.309	−4.908	132.90	296.41	−1.762
MNPD13725	456.5	Nil	0	8	92.27 (High)	4.145	−4.915	85.41	196.91	−2.013

### 3.4 Toxicity analysis

Evaluation of toxicity reveals a spectrum of risks and safety profiles associated with lead molecules, pivotal to determining their therapeutic suitability. The ProTox-II web server, was employed to comprehensively assess the toxicity profiles of the identified molecules. The ProTox-II server utilizes advanced computational models to predict the toxicity of compounds based on 2D similarity analysis between the molecules under scrutiny and those already present in its extensive library, which includes compounds with established LD_50_ values. This approach provides valuable insights into potential toxicity risks by comparing structural and chemical properties. The ProTox-II server divides chemical compounds into various toxicity classes according to the Globally Harmonized System regulations, ranging up to Class VI, based on their LD_50_ values (mg/kg body weight). The detailed LD_50_ values of the identified MNPs are listed in S1 Table. Notably, three MNPs from the CMNPD (CMNPD4682, CMNPD14329, and CMNPD23880) were classified as Class V, indicating a low likelihood of toxicity. In contrast, the remaining two MNPs, CMNPD28977 and CMNPD24734, fell into Class IV, suggesting that they are harmful if ingested. Similarly, compounds from the MNPD, such as MNPD9492, MNPD9493, and MNPD13725, were classified as Class IV with LD50 values of 555, 555, and 841 mg/kg, respectively. MNPD9355 was classified as a low toxicity compound with an LD_50_ value of 3000 mg/kg, whereas MNPD13399 was categorized as a toxic compound with a low toxicity classification in Class II. These classifications and LD_50_ values provide a clear understanding of the potential risks associated with these compounds. Additionally, as shown in S1 Table, all the identified MNPs were expected to exhibit no hepatotoxicity, cytotoxicity, carcinogenicity, mutagenicity, or immunotoxicity. This comprehensive safety profile indicates that the selected MNPs are safe for biological use. Furthermore, their lack of toxicity, coupled with their potential efficacy, suggests that these MNPs could be promising candidates for the development of anti-biofilm inhibitors.

### 3.5 Frontier molecular orbital analysis

**Fig 2:**
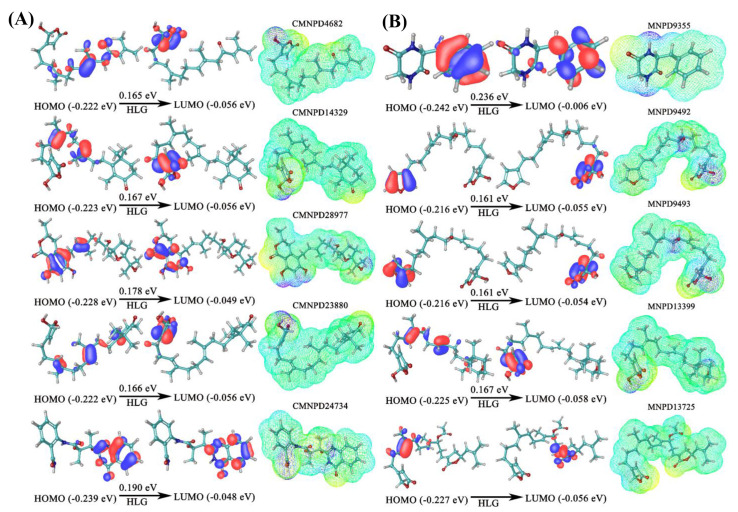
Frontier molecular orbital analysis of identified marine natural products (MNPs) from the CMNPD (A) and MNPD (B) databases, including representations of HOMO, LUMO, and MESP maps. The red and blue regions in the molecular orbital diagrams indicate positive and negative phase distributions, respectively. The MESP map, shown in a rainbow format, highlights electron-rich and electron-poor regions on the molecular surface.

The study of FMOs is essential for gaining a comprehensive understanding of molecular reactivity and stability. A key aspect of this analysis is the pivotal role of the outermost electrons in facilitating interactions between the chemical scaffolds and target protein. In particular, examining the HOMO and LUMO provide deeper insights into compound chemical reactivity. The HOMO indicates a molecule’s propensity to donate electrons, while the LUMO shows its ability to accept electrons. The energy gap between these orbitals, known as the orbital energy gap, highlights efficient charge transfer mechanisms and enhances molecule polarizability. The FMO analysis was used to predict the most reactive regions within the identified MNPs. The HOMO and LUMO energies of the MNPs and their respective orbital energies are listed in Table 3. Fig 2 provide visual representations of the most reactive regions in these compounds, with red and blue indicating the positive and negative phase distributions in the molecular orbital wave function, respectively. Understanding the localization of the HOMO and LUMO in a ligand is crucial as it provides insights into how the molecule interacts with a target receptor.

**Table 3:** Frontier molecular orbital analysis of MNPs identified *via* virtual screening using DFT calculation.

MNPs	HOMO energy (eV)	LUMO energy (eV)	Energy Gap (eV)	MESP (kcal/mol)	
**Most negative potential**	**Most positive potential**
CMNPD4682	−0.2221	−0.0564	0.165	−58.13	325.60
CMNPD14329	−0.2235	−0.0563	0.167	−53.75	410.68
CMNPD28977	−0.2281	−0.0498	0.178	−66.38	393.61
CMNPD23880	−0.2225	−0.0561	0.166	−54.56	399.69
CMNPD24734	−0.2390	−0.0487	0.190	−59.39	346.39
MNPD9355	−0.2422	−0.0061	0.236	−46.89	208.28
MNPD9492	−0.2165	−0.0551	0.161	−54.53	341.78
MNPD9493	−0.2164	−0.0547	0.161	−54.62	342.20
MNPD13399	−0.2252	−0.0580	0.167	−54.46	400.83
MNPD13725	−0.2278	−0.0567	0.171	−63.40	422.40

In CMNPD4682, the HOMO is localized within the functional group, whereas the LUMO occupied the furan ring, resulting in an orbital energy gap of 0.165 eV (Fig 2A). In CMNPD14329, the HOMO was predominantly associated with the carbon atoms in the functional group, whereas the LUMO was distributed across the ring carbon and oxygen atoms. For CMNPD28977, both the HOMO and LUMO were occupied by the aromatic ring carbon and oxygen atoms. For CMNPD23880, the HOMO was dispersed across the functional groups of the molecule, whereas the LUMO was localized in the carbonyl (C=O) and hydroxyl (-OH) groups attached to the furan ring. Both the HOMO and LUMO in CMNPD24734 were occupied within the fused six-membered aromatic rings, specifically the benzene and pyrimidine rings. Among the MNPs, CMNPD24734 exhibited the highest HOMO energy and largest orbital energy gap, indicating its high chemical stability and significant electron-donating capability. This high electron-donating capability suggests strong functional group interactions with binding site residues within the PqsR protein. Thus, the results of the DFT calculations bolster the findings of the docking-based screening, providing a comprehensive understanding of the molecular interactions and reinforcing the potential efficacy of these MNPs.

The results of FMO analysis conducted on the MNPs obtained from the MNPD, are illustrated in Fig 2B. This analysis revealed that, in MNPD9355, both the HOMO and LUMO were predominantly localized within the benzene ring. This localization suggests strong electron interactions within the ring structure. Additionally, the high orbital energy gap score of 0.236 eV observed for MNPD9355 indicated a significant electron transfer process, highlighting the potential of the molecule for high chemical stability and reactivity in molecular interactions. Compounds MNPD9492 and MNPD9493, which have similar scaffolds, also showed HOMO and LUMO localization at the aromatic ring carbon and oxygen atoms, with an orbital energy gap of 0.161 eV. For MNPD13399 and MNPD13725, the HOMO was distributed over the functional group carbon atoms, whereas the LUMO occupied the aromatic ring atoms. This distribution suggests that the specific electronic properties and potential reactivity patterns are unique to each compound, emphasizing their potential roles in molecular interactions.

The MESP map, which is a 3D representation of charge distribution on the molecules, was plotted using the 6-31G^**++^ basis set. In recent years, MESP has become a powerful tool for exploring molecular interactions, offering valuable insights into electrostatic properties and reactive sites. The MESP plot shows various regions using distinct colors. Red and yellow surfaces indicate areas of high electron density correlating with nucleophilic sites, whereas blue signifies regions of low electron density linked to electrophilic sites. The green surfaces denote regions with zero potential. For the molecules sourced from the CMNPD, the most negative electrostatic potential value was observed for CMNPD28977 (−66.38 kcal/mol), and the most positive for CMNPD14329 (410.68 kcal/mol). Similarly, among the molecules obtained from the MNPD, MNPD13725 exhibited the highest negative (−63.40 kcal/mol) and positive (422.40 kcal/mol) electrostatic potential values. The MESP analysis revealed that the atoms involved in hydrogen bond interactions were surrounded by both positive and negative electrostatic potentials, which is consistent with the molecular interaction profiles of the identified molecules.

### 3.6 Mechanistic insights into marine natural product structural stability with PqsR

**Fig 3:**
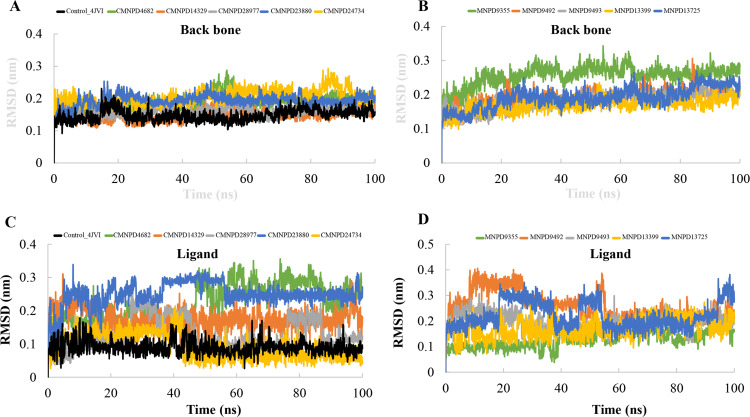
Molecular dynamic trajectory analysis: Backbone (A, B) and Ligand (C, D) RMSD of all the complexes.

**Fig 4:**
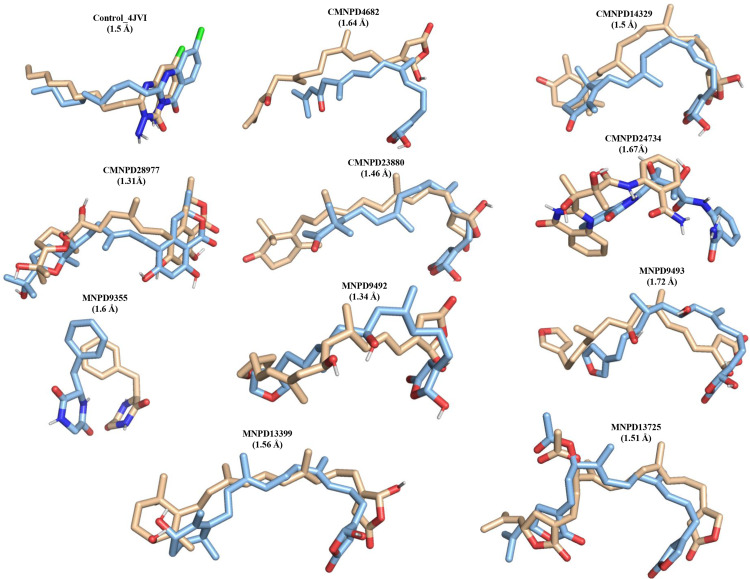
The superimposition of the initial (light blue color) and low energy (wheat) complexes were performed to show the ligand deviation from the binding pocket. The RMSD between the initial and final complexes are given in brackets. For clarity, only ligands are shown.

To gain mechanistic insights into the structural stability of MNPs complexed with PqsR, we performed MD simulations on a 100 ns time scale. The structural dynamics of the complexes based on the initial qualitative analysis were depicted in Fig 3. Using the MD trajectories, we calculated the time-dependent RMSDs of the backbone atoms with respect to the initial structure. The RMSD analyses for all systems bound to CMNPD and MNPD molecules are depicted in Figs 3A and 3B, respectively. The backbone RMSD plots revealed a consistent trend across all the complexes, demonstrating a stable equilibrated pattern, with the RMSD values remaining below ~0.35 nm. This stability was observed in both the control and identified lead molecule-bound complexes, indicating strong structural integrity throughout the simulations. Next, the structural stability of the MNPs within the binding pocket of PqsR was assessed by calculating the ligand RMSD (Figs 3C and 3D). The RMSD values, which were consistently below 0.4 nm, demonstrated the strong binding affinity of the MNPs in the binding pocket throughout the simulation. A similar RMSD trend was observed for both the control and CMNPD24734, with their RMSD values being nearly identical, indicating comparable stability and binding behavior. Specifically, CMNPD4682 and CMNPD23880 exhibited the maximum positional deviations (>0.25 nm) within the binding pocket of PqsR, with two distinct equilibration phases observed during the simulation. In contrast, CMNPD14329 showed a consistent equilibration pattern and the highest stability, with RMSD values remaining below approximately 0.2 nm. Additionally, ligand superimposition of the initial and low-energy structures for each complex of the trajectory is illustrated in Fig 4, which suggest that the MNPs are not largely deviated or move out from the binding pocket, however, the initial conformation is slightly varied within the binding pocket due to the size of the pocket. Overall the above results corroborating the conformational stability of the MNP-bound PqsR complexes.

The structural compactness of all MNP-bound complexes was subsequently monitored by calculating Rg throughout the production simulation, as illustrated in S3 Fig. The average Rg for complexes containing CMNPD molecules with PqsR ranged from ~1.77 nm to 1.84 nm, and varied from ~1.77 nm to 1.86 nm for complexes containing MNPD molecules. This consistent range of Rg values indicated that the binding of MNPs did not induce any significant changes in the structural compactness of the complexes. To further complement the stability analysis H-bond interactions between PqsR and the identified MNPs were examined throughout the simulation. The number of H-bonds formed was computed to provide additional insight into the stability of the protein-ligand complexes. A comprehensive analysis of the prominent H-bonds is illustrated in S4 Fig and S5 Fig, which highlights the crucial role of these interactions in maintaining the structural integrity of the complexes. It is evident that all the MNP-bound complexes exhibited a significantly higher number of H-bonds than the control molecule. This increased number of H-bonds enhanced the electrostatic interactions between PqsR and the MNPs, contributing to the overall stability and robustness of the protein-ligand complexes.

### 3.7 Energetics of marine natural products binding affinity

To explore the efficacy of the identified MNPs against PqsR, MM/PBSA-based calculations were performed to assess their BFEs, offering valuable insights into ligand affinity and elucidating the specific energetic contributions that facilitate binding. We identified key residues essential for stabilizing protein-ligand complexes, enhancing our understanding of their interaction dynamics. A comprehensive summary of the BFE interaction profile and its energy components is presented in Table 4. As shown, the polar solvation energy (ΔG_pol_) disfavors the binding of MNPs. In contrast, the other energy components, including van der Waals energy (ΔG_vdW_), electrostatic energy (ΔG_elec_), and solvent-accessible surface area energy, are favorable towards protein-ligand complexes. Notably, the van der Waals energy (ΔG_vdW_) is the dominant factor, contributing significantly to the total binding free energy. Further analysis of the BFE profile revealed that the calculated binding affinities of CMNPD14329, CMNPD23880, MNPD13399, and MNPD13725 to PqsR were significantly higher than those of the control molecule and other MNP-bound complexes, suggesting that these specific MNPs formed more stable interactions with PqsR. In contrast, MNPD9355 exhibited the lowest binding affinity (−39.44 +/- 9.68 kJ/mol) within the complexes, attributed to its low van der Waals energy component, indicating it is not an effective binder against PqsR. These results were corroborated by docking and MM/GBSA binding energy scores (Table 1). Notably, molecules MNPD13725 and CMNPD23880 demonstrated the highest binding affinity scores of −148.10 +/- 11.82 kJ/mol and −137.85 +/- 14.61 kJ/mol, respectively. These findings highlight CMNPD23880 and MNPD13725 as promising candidates for targeting PqsR.

**Table 4:** MM/PBSA-derived energetic components of BFE for PqsR of *Pseudomonas aeruginosa* in complex with MNPs.

Complexes	Energy Components				
**Van der Waal energy (kJ/mol)**	**Electrostatic energy (kJ/mol)**	**Polar solvation energy (kJ/mol)**	**SASA energy (kJ/mol)**	**Binding energy (kJ/mol)**
Control_4JVI	−168.49 +/- 9.88	−14.53 +/- 6.2	83.60 +/- 8.87	−19.37 +/- 0.91	−118.78 +/- 10.6
CMNPD4682	−153.53 +/- 15.46	−7.95 +/- 8.16	89.68 +/- 14.63	−21.01 +/- 1.34	−92.81 +/- 12.54
CMNPD14329	−194.37 +/- 9.97	−17.52 +/- 7.59	113.31 +/- 9.05	−22.65 +/- 0.91	−121.24 +/- 11.65
CMNPD28977	−197.64 +/- 10.55	−13.64 +/- 7.16	124.57 +/- 10.80	−22.44 +/- 1.00	−109.14 +/- 11.22
CMNPD23880	−198.75 +/- 11.30	−26.99 +/- 8.84	111.81 +/- 20.68	−23.92 +/- 0.99	−137.85 +/- 14.61
CMNPD24734	−136.37 +/- 11.61	−36.70 +/- 9.45	111.17 +/- 15.51	−16.54 +/- 1.11	−78.44 +/- 10.47
MNPD9355	−96.47 +/- 8.94	−27.18 +/- 10.82	96.91 +/- 12.43	−12.70 +/- 0.74	−39.44 +/- 9.68
MNPD9492	−180.28 +/- 10.80	−19.40 +/- 7.87	121.50 +/- 11.87	−22.60 +/- 0.88	−100.79 +/- 11.45
MNPD9493	−174.70 +/- 15.03	−26.47 +/- 9.00	125.31 +/- 17.02	−22.37 +/- 0.88	−98.24 +/- 12.46
MNPD13399	−202.31 +/- 10.98	−29.49 +/- 8.71	120.93 +/- 15.78	−24.88 +/- 0.89	−135.76 +/- 14.22
MNPD13725	−220.23 +/- 12.14	−41.10 +/- 9.71	140.60 +/- 11.76	−27.36 +/- 1.00	−148.10 +/- 11.82

Note: SASA, solvent-accessible surface area.

**Fig 5:**
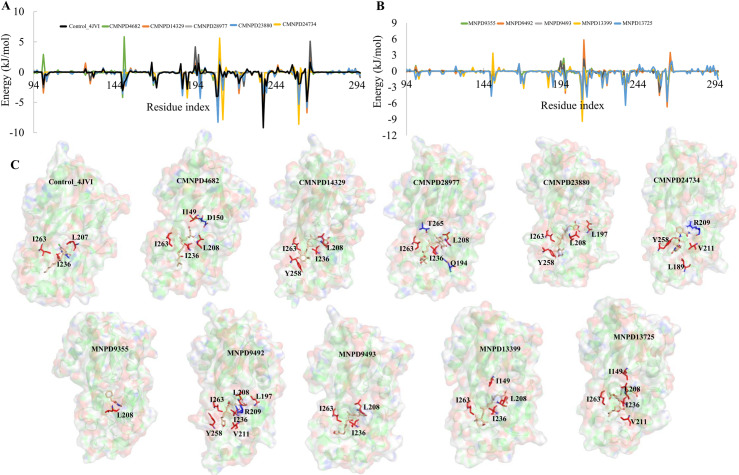
Energy decomposition. Per residue energy decomposition shown for all the complexes (A) CMNPD, (B) MNPD, (C) The hot-spot residues maintaining the criteria of greater than ±4 are identified on the structure. Residues with positive values are shown in blue and negative are shown in red.

A per-residue energy decomposition analysis was conducted to identify hot-spot residues, defined as those contributing more than −4 kcal/mol to the binding energy, which are critical for strong binding interactions with PqsR. Energy decomposition plots (Fig 5A and 5B) highlight the essential residues within the complexes. Additionally, their specific positions within the binding pocket provided a detailed map of the key interaction sites responsible for enhanced binding affinity (Fig 5C). It is evident that residues L208 and I263 are favorable hot-spot residues commonly present in PqsR complexes with CMNPD4682, CMNPD14329, CMNPD28977, and CMNPD23880. For CMNPD24734, four favorable binding residues (L189, R209, V211, and Y258) were identified as crucial for maintaining structural stability within the complex. Similarly, in all complexes except for MNPD9355, three hot-spot residues (L208, I236, and I263) predominantly contributed to the binding energy profile, significantly enhancing the structural stability of the complexes. Van der Waals interactions with these residues play a vital role in the overall binding affinity of the MNPs. In contrast, only a single hot-spot residue (L208) was identified in the PqsR-MNPD9355 complex, further confirming the weak binding of MNPD9355 to the complexes. These findings underscore the importance of hot-spot residues in enhancing the binding affinity and stability of MNPs to PqsR.

## 4 Conclusion

In summary, this study aimed to counter antimicrobial resistance by exploring potential MNPs from two different databases to identify promising lead molecules targeting the biofilm-forming protein PqsR from *P. aeruginosa*. Using hierarchical virtual screening methodologies and structural dynamics studies, we identified several promising candidates as effective lead molecules against this resilient pathogen. Among the ten identified lead molecules, CMNPD14329, CMNPD23880, MNPD13399 and MNPD13725 stood out as potential compounds for future research. These molecules can serve as the basis for developing structural analogs with enhanced binding affinities for PqsR and other biofilm-forming proteins. However, it is crucial to note that the computational findings of this study require further experimental validation to confirm the efficacy of the identified lead molecules in inhibiting biofilm formation. Future studies should focus on rigorous *in vitro* and *in vivo* testing to ensure that these candidates effectively combat *P. aeruginosa* and contribute to controlling antimicrobial resistance.

## Supplementary information

S1 Fig Crystal structure of the PqsR co-inducer binding domain from Pseudomonas aeruginosa complexed with an inhibitor (PDB ID: 4JVI). The enlarged view highlights the interactions between the inhibitor and the binding pocket residues of PqsR. (TIF)

S2 Fig 2D-chemical structures of identified hit-molecules from the structure based virtual screening (SBVS) approach. (TIF)

S3 Fig The radius of gyration of all the complexes. (TIF)

S4 Fig The total number of hydrogen bonds observed between CMNPD and PqsR. (TIF)

S5 Fig The total number of hydrogen bonds observed between MNPD and PqsR. (TIF)

S1 Table Predicted toxicity properties of virtual screening identified MNPs through ProTox-II server. (DOCX)

S2 Table Top-ranked marine natural products (MNPs) selected based on stringent assessment criteria, including docking scores, MM-GBSA binding energy, and pharmacokinetic property predictions using the QikProp module of Schrödinger software. (DOCX)
