# Supplementary figures and images for: Exploring Marine natural products as potential Quorum sensing inhibitors by targeting the PqsR in Pseudomonas aeruginosa: Virtual screening assisted structural dynamics study
Source: PLoS One. 2025 Mar 28;20(3):e0319352. doi: 10.1371/journal.pone.0319352 (PMC11952224; doi:10.1371/journal.pone.0319352)

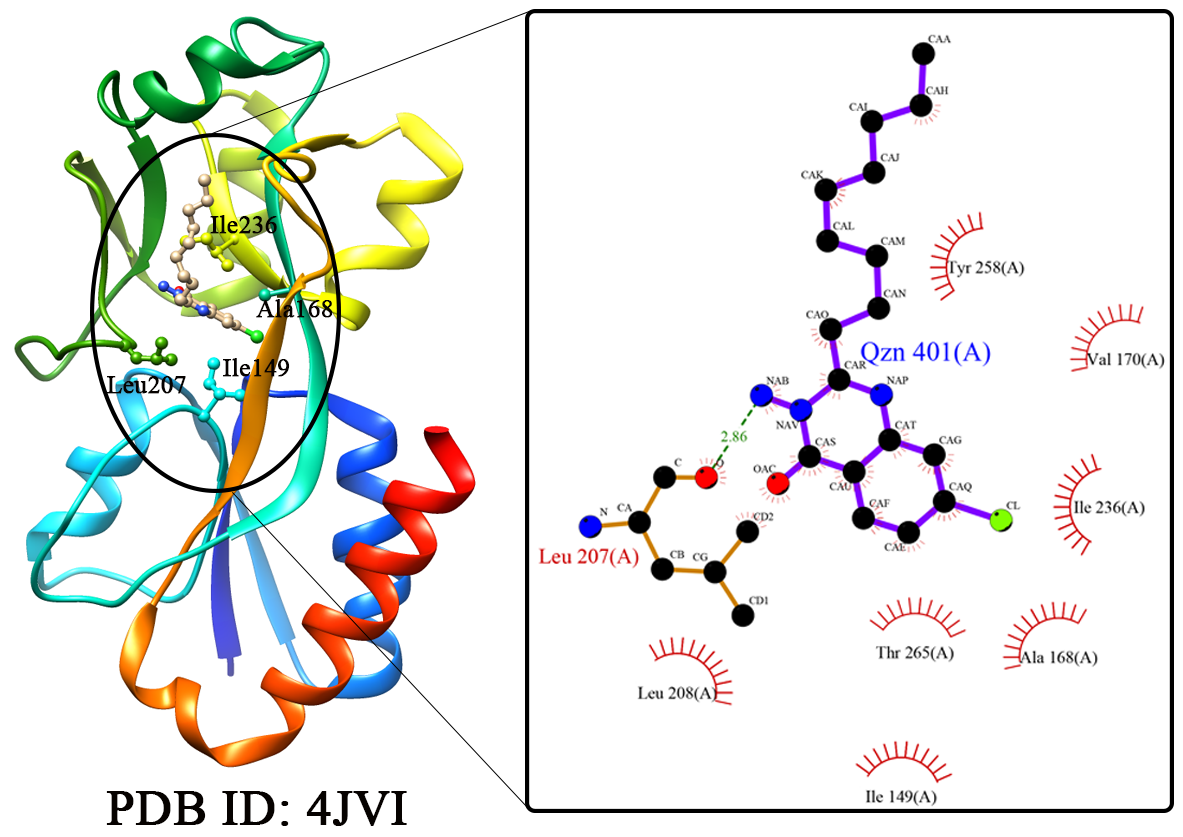

Supplement: S1 Fig — (TIF) [file pone.0319352.s001.tif]

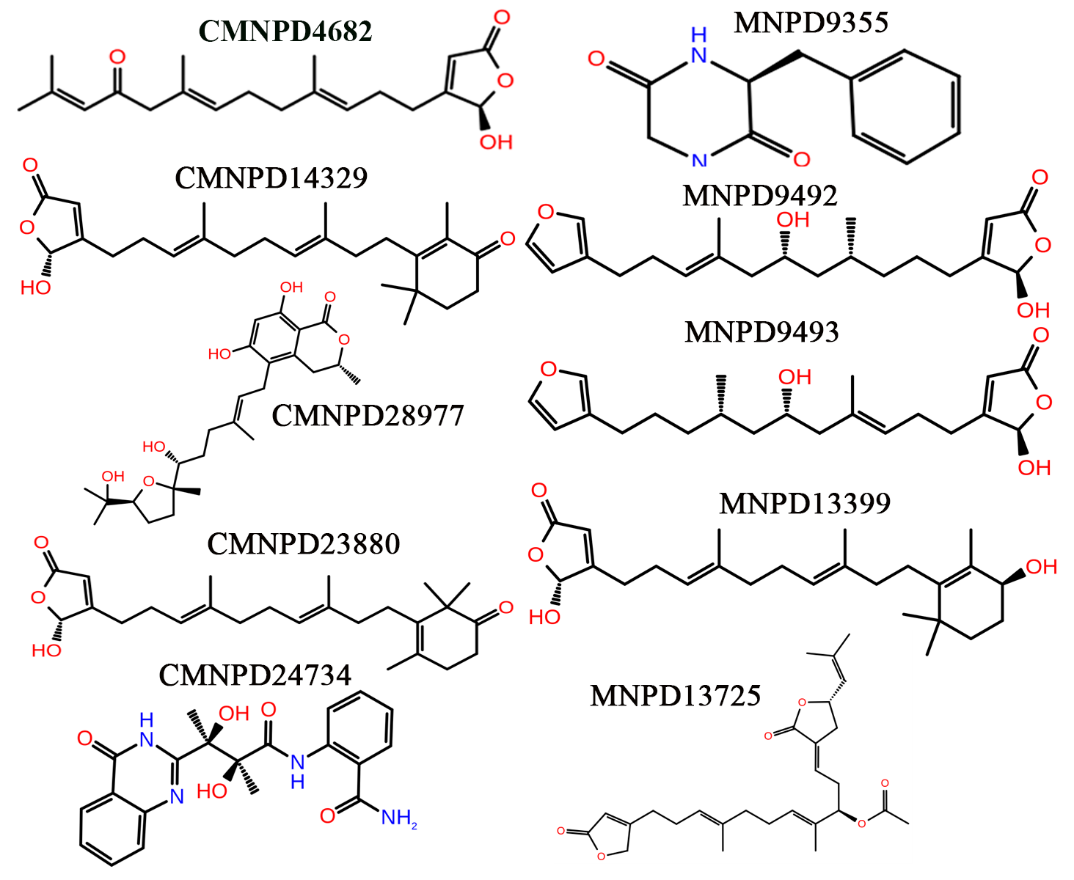

Supplement: S2 Fig — (TIF) [file pone.0319352.s002.tif]

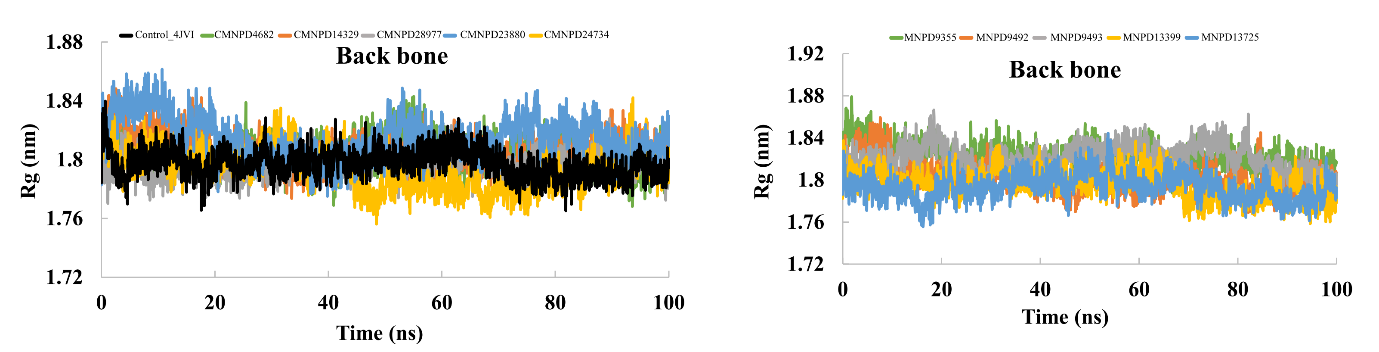

Supplement: S3 Fig — (TIF) [file pone.0319352.s003.tif]

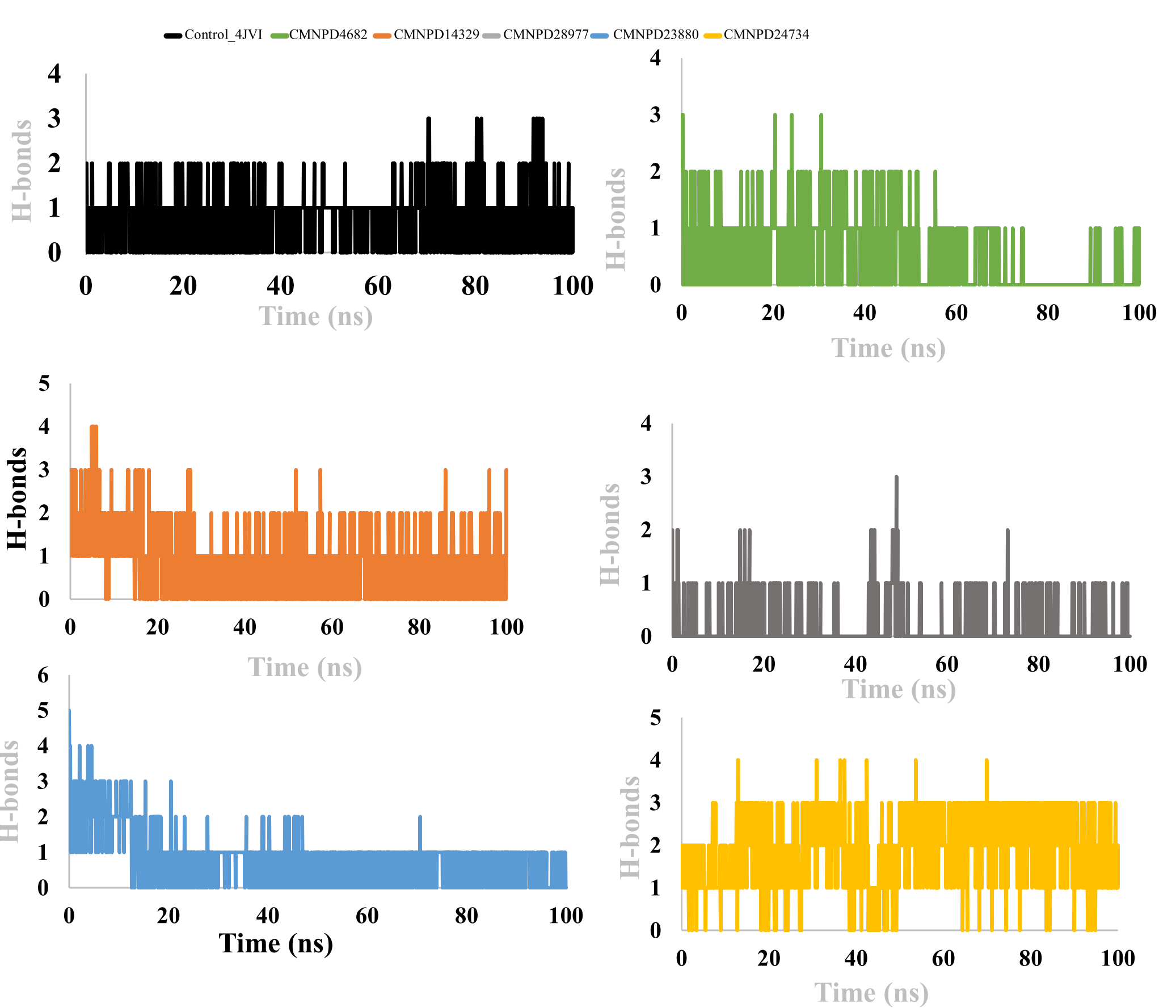

Supplement: S4 Fig — (TIF) [file pone.0319352.s004.tif]

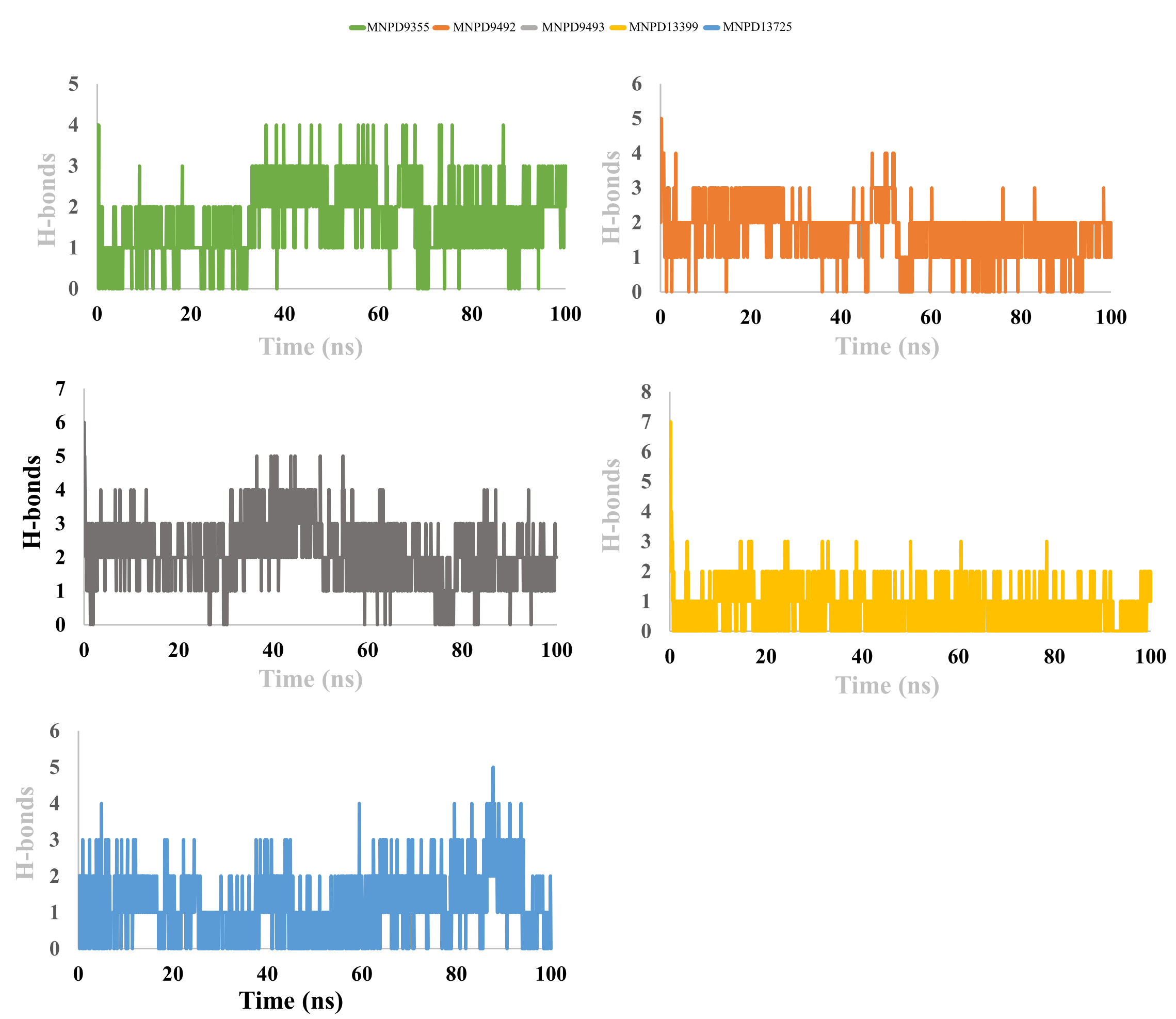

Supplement: S5 Fig — (TIF) [file pone.0319352.s005.tif]
