# Supplementary material for: Exploring Marine natural products as potential Quorum sensing inhibitors by targeting the PqsR in Pseudomonas aeruginosa: Virtual screening assisted structural dynamics study
Source: PLoS One. 2025 Mar 28;20(3):e0319352. doi: 10.1371/journal.pone.0319352 (PMC11952224; doi:10.1371/journal.pone.0319352)
Supplement: S1 Table — (DOCX) [file pone.0319352.s006.docx]

**S1 Table.** Predicted toxicity properties of virtual screening identified MNPs through ProTox-II server.

| **Marine Natural Products** | **ProTox-II Class** | **LD_50_ values**  **(mg/kg)** | **Hepatotoxicity** | **Cytotoxicity** | **Carcinogenicity** | **Mutagenicity** | **Immunotoxicity** |
| --- | --- | --- | --- | --- | --- | --- | --- |
| **CMNPD** | | | | | | | |
| CMNPD4682 | 5 | 5000 | Inactive (0.79) | Inactive (0.63) | Inactive (0.63) | Inactive (0.82) | Inactive (0.96) |
| CMNPD14329 | 5 | 5000 | Inactive (0.80) | Inactive (0.64) | Inactive (0.65) | Inactive (0.75) | Inactive (0.62) |
| CMNPD28977 | 4 | 1000 | Inactive (0.86) | Inactive (0.69) | Inactive (0.63) | Inactive (0.75) | Inactive (0.62) |
| CMNPD23880 | 5 | 5000 | Inactive (0.80) | Inactive (0.64) | Inactive (0.65) | Inactive (0.75) | Inactive (0.65) |
| CMNPD24734 | 4 | 1250 | Inactive (0.62) | Inactive (0.66) | Inactive (0.51) | Inactive (0.64) | Inactive (0.99) |
| **MNPD** | | | | | | | |
| MNPD9355 | 5 | 3000 | Inactive (0.85) | Inactive (0.81) | Inactive (0.66) | Inactive (0.77) | Inactive (0.99) |
| MNPD9492 | 4 | 555 | Inactive (0.86) | Inactive (0.64) | Inactive (0.69) | Inactive (0.83) | Inactive (0.54) |
| MNPD9493 | 4 | 555 | Inactive (0.86) | Active (0.64) | Inactive (0.69) | Inactive (0.83) | Inactive (0.55) |
| NPD13399 | 2 | 34 | Inactive (0.79) | Inactive (0.65) | Inactive (0.64) | Inactive (0.69) | Inactive (0.55) |
| MNPD13725 | 4 | 841 | Inactive (0.81) | Inactive (0.64) | Inactive (0.62) | Inactive (0.84) | Inactive (0.99) |
