# Supplementary material for: Exploring Marine natural products as potential Quorum sensing inhibitors by targeting the PqsR in Pseudomonas aeruginosa: Virtual screening assisted structural dynamics study
Source: PLoS One. 2025 Mar 28;20(3):e0319352. doi: 10.1371/journal.pone.0319352 (PMC11952224; doi:10.1371/journal.pone.0319352)
Supplement: S2 Table — (DOCX) [file pone.0319352.s007.docx]

**S2 Table.** Top-ranked marine natural products (MNPs) selected based on stringent assessment criteria, including docking scores, MM-GBSA binding energy, and pharmacokinetic property predictions using the QikProp module of Schrödinger software.

| **S. No** | **MNPs** | **Docking Score** | **MMGBSA** | **MW** | **Ro5 viol** | **Donor**  **HB** | **Accpt**  **HB** | **HOA (%)** | **QPlogPo/w** | **QPlogHERG** | **QPPMDCK** | **QPPCaco** | **QPlogBB** |  |
| --- | --- | --- | --- | --- | --- | --- | --- | --- | --- | --- | --- | --- | --- | --- |
| **CMNPD** | | | | | | | | | | | | | | |
| 01 | CMNPD24734 | −13.08 | −56.29 | 396.4 | Nil | 4 | 8 | 63.06 | 1.856 | −5.460 | 57.43 | 136.40 | −1.678 |  |
| 02 | CMNPD23880 | −12.60 | −60.41 | 400.5 | Nil | 1 | 6.7 | 100 | 4.343 | −4.931 | 209.46 | 451.53 | −1.504 |  |
| 03 | CMNPD28977 | −11.85 | −52.67 | 448.5 | Nil | 3 | 6.7 | 90.13 | 3.889 | −4.635 | 78.11 | 181.28 | −1.888 |  |
| 04 | CMNPD14329 | −11.80 | −55.55 | 400.5 | Nil | 1 | 6.7 | 95.17 | 4.173 | −4.818 | 124.91 | 279.88 | −1.722 |  |
| 05 | CMNPD4682 | −11.59 | −56.98 | 332.4 | Nil | 1 | 6.7 | 91.24 | 3.13 | −4.835 | 168.94 | 370.01 | −1.558 |  |
| 06 | CMNPD14332 | −11.58 | −43.13 | 418.5 | Nil | 1 | 7.4 | 92.82 | 4.007 | −5.025 | 103.113 | 234.382 | −1.976 |  |
| 07 | CMNPD17835 | −11.42 | −48.64 | 323.1 | Nil | 4 | 3 | 77.61 | 1.647 | −4.238 | 213.451 | 195.931 | −1.071 |  |
| 08 | CMNPD10438 | −11.36 | −54.21 | 359.1 | Nil | 4 | 6.95 | 75.35 | 1.024 | −4.453 | 288.006 | 234.273 | −1.071 |  |
| 09 | CMNPD24733 | −11.41 | −56.32 | 396.4 | 1 | 4 | 8 | 55.28 | 1.697 | −5.826 | 22.166 | 56.527 | −2.182 |  |
| 10 | CMNPD2739 | −11.09 | −45.30 | 342.3 | Nil | 3 | 3.25 | 84.58 | 2.988 | −5.523 | 75.208 | 175.038 | −1.266 |  |
| **MNPD** | | | | | | | | | | | | | | |
| 01 | MNPD9492 | −13.07 | −64.51 | 362.4 | Nil | 2 | 6.9 | 92.87 | 3.387 | −5.793 | 171.97 | 376.23 | −1.810 |  |
| 02 | MNPD9493 | −12.68 | −64.53 | 362.4 | Nil | 2 | 6.9 | 92.20 | 3.359 | −5.765 | 160.43 | 352.80 | −1.834 |  |
| 03 | MNPD9355 | −12.57 | −43.13 | 204.2 | Nil | 2 | 5 | 62.61 | -0.481 | −0.718 | 270.56 | 141.29 | −0.560 |  |
| 04 | MNPD13399 | −12.44 | −64.37 | 402.5 | Nil | 2 | 6.4 | 96.41 | 4.309 | −4.908 | 132.90 | 296.41 | −1.762 |  |
| 05 | MNPD13725 | −11.88 | −56.80 | 456.5 | Nil | 0 | 8 | 92.27 | 4.145 | −4.915 | 85.41 | 196.91 | −2.013 |  |
| 06 | MNPD6967 | −11.38 | −53.94 | 359.17 | 1 | 4 | 6.95 | 73.86 | 1.029 | −4.769 | 231.35 | 192.76 | −1.232 |  |
| 07 | MNPD4439 | −11.24 | −67.10 | 471.59 | Nil | 2 | 8.9 | 84.68 | 3.974 | −5.645 | 34.151 | 84.31 | −2.689 |  |
| 08 | MNPD9947 | −11.15 | −41.42 | 234.25 | Nil | 3 | 5.75 | 55.60 | −0.782 | −0.548 | 112.53 | 71.91 | −0.911 |  |
| 09 | MNPD12080 | −10.93 | −54.92 | 416.55 | 1 | 2 | 8.1 | 100 | 3.427 | −4.217 | 302.51 | 634.42 | −1.089 |  |
| 10 | MNPD6404 | −10.59 | −46.28 | 257.24 | Nil | 4 | 9.8 | 53.25 | -1.469 | −3.88 | 36.304 | 89.22 | −1.599 |  |
